# Comparative Study on Intermediate-Temperature Deformation Mechanisms of Inconel 718 Alloys Fabricated by Additive Manufacturing and Conventional Forging

**DOI:** 10.3390/ma18235354

**Published:** 2025-11-27

**Authors:** Jin Wu, Yetao Cheng, Jinlong Su, Yubin Ke, Jie Teng, Fulin Jiang

**Affiliations:** 1 State Key Laboratory of Cemented Carbide, College of Materials Science and Engineering, Hunan University, Changsha 410082, China; 2 Beihai Petrochemical and New Materials Industry Development Promotion Center, Beihai 536000, China; 3 Department of Mechanical Engineering, City University of Hong Kong, Kowloon, Hong Kong SAR, China; 4 Spallation Neutron Source Science Center, Dongguan 523803, China; 5 Institute of High Energy Physics, Chinese Academy of Sciences, Beijing 100049, China

**Keywords:** Inconel 718 Alloy, additive manufacturing, microstructure, intermediate temperature deformation behavior

## Abstract

The distinct solidification behavior of additively manufactured (AM) Inconel 718 (IN718) produces a unique microstructure and precipitation response compared with its conventionally forged counterpart, leading to fundamentally different responses to heat treatment and intermediate-temperature deformation behaviors. In this work, the intermediate-temperature (450–750 °C) deformation mechanisms of laser powder bed fusion (LPBF)-fabricated and forged IN718 alloys were systematically compared under various heat-treatment conditions. Overall, under solution treatment state, the LPBF alloy exhibited fine columnar grains, a high dislocation density, and retained δ phases along the grain boundaries, whereas the forged alloy showed coarse equiaxed γ grains without the δ phase. Under solution + aging (STA) treatment, the δ phase in the LPBF alloy effectively pinned grain boundaries and enhanced flow stress, while in the forged alloy, strengthening was dominated by the uniform precipitation of γ″ and γ′ phases. Owing to Nb consumption by δ-phase formation, the STA-treated LPBF alloy contained fewer γ″/γ′ precipitates and exhibited slightly lower strength than the STA-treated forged alloy. This study demonstrates that the inherent δ phase retention and Nb segregation in LPBF-built IN718 critically influence its precipitation behavior and deformation resistance, distinguishing it from conventionally processed alloys and providing valuable insights for microstructure design in AM-built high-temperature superalloys.

## 1. Introduction

Nickel-based superalloys are widely used in demanding structural applications operating at intermediate to high temperatures due to their outstanding mechanical strength, oxidation, and corrosion resistance [1,2]. Among them, the Inconel 718 (IN718) alloy, known as the “king of superalloy in terms of performance”, is the most widely used deformable superalloy, accounting for more than 50% of the total consumption of deformable alloys [3,4]. The IN718 alloy is an age-hardening alloy with an austenitic (γ) matrix phase and strengthening phases γ′-Ni_3_(Al, Ti), γ″-Ni_3_Nb, and δ [5,6]. The main strengthening mechanism of IN718 alloy is precipitation strengthening, and the size, number density, and distribution of precipitates determine its strengthening effects [7,8]. It exhibits excellent high-temperature strength and oxidation resistance and is widely employed in the aerospace and nuclear sectors—for instance, in rocket engine components, turbine disks of aero-engines, and fuel assemblies of water-cooled nuclear reactions [9,10,11,12]. Within its typical operating temperature range of 450–650 °C, the IN718 alloy exhibits anomalous strengthening and a marked decrease in plasticity during intermediate-temperature deformation [13].

The typical heat treatment routes of IN718 comprise solution treatment (ST) and solution + aging treatment (STA) [14]. Solution treatment can be used to obtain a uniform grain size, dissolve the Laves phase into the matrix, and obtain a single austenitic phase, laying the foundation for the precipitation in the subsequent aging treatment. Solution + aging treatment can induce the precipitation of strengthening phases, such as γ″, γ′, and δ precipitate, thereby achieving a sufficient precipitation strengthening effect. The precipitation temperature of the δ phase ranges from 780 °C to 980 °C [15]. When aging, treated above 980 °C, the δ phase begins to dissolve [16]. Notably, the δ phase has a dual effect on the mechanical properties of the alloy. Firstly, it shares a similar composition with γ″ and γ′, leading to competitive precipitation behavior [17]. This is to say that excessive δ phase precipitation will consume Nb and reduce γ″/γ′ formation, degrading strength, creep resistance, and rupture life. Nevertheless, a moderate amount of the δ phase is considered to enhance ductility, reduce notch sensitivity, and alleviate local stress concentration [18].

Traditionally, IN718 alloy components are produced via casting, forging, or extrusion, with forging being the most common route [19]. However, owing to the high hardness, large cutting force, and low thermal conductivity of Ni-based alloys, their machining imposes stringent requirements on the processing equipment. At the same time, defects and element segregation are easy to cause during the casting process, making the traditional manufacturing processes time-consuming and labor-intensive [20]. Driven by the increasing need for intricate and highly integrated alloy structures, additive manufacturing (AM) has emerged as a particularly advantageous fabrication technology [21,22]. AM is a rapid prototyping technology characterized by the “accumulation from scattered parts”, “top-down”, “creation from nothing”, and high efficiency [16]. Among AM technologies, laser powder bed fusion (LPBF) is particularly suited for metals and alloys, producing dense components with refined microstructures and high geometric accuracy [23,24,25,26]. Due to its relatively low (Al + Ti) content, IN718 alloy exhibits excellent weldability and is less susceptible to solidification cracking during LPBF processing [27].

Given that the IN718 alloy is typically employed at service temperatures between 450 °C and 650 °C, understanding its deformation mechanisms in the intermediate-temperature region is critical for ensuring reliable performance in practical applications. In conventionally forged IN718, the δ phase preferentially precipitates along grain boundaries, where it impedes dislocation motion and consequently enhances strength at intermediate temperatures [28]. In contrast, laser powder bed fusion (LPBF)-fabricated IN718 undergoes rapid solidification kinetics during subsequent heat treatment [29,30]. Despite these pronounced microstructural differences between forged and additively manufactured counterparts, systematic investigations into the intermediate-temperature deformation behavior of LPBF-built IN718 alloys remain scarce [31,32]. Addressing this knowledge gap is essential for establishing a mechanistic understanding that links δ phase evolution to deformation response, thereby guiding the optimization of heat treatment and service performance of AM superalloys.

In this paper, the intermediate-temperature deformation mechanisms (450–750 °C) of LPBF-built and conventionally forged IN718 alloys were systematically compared under various heat-treatment conditions (i.e., ST and STA). Through combined intermediate-temperature compression testing, electron backscatter diffraction (EBSD), and transmission electron microscopy (TEM) analyses, the evolutions of strengthening phases, dislocation behavior, and deformation microstructures were investigated systematically. This work aims to clarify how distinct precipitation responses and δ phase characteristics govern the intermediate-temperature deformation behavior of AM and forged IN718 alloys, providing insights for optimizing the heat treatment and service performance of AM superalloys.

## 2. Materials and Methods

### 2.1. Materials

**Figure 1:**
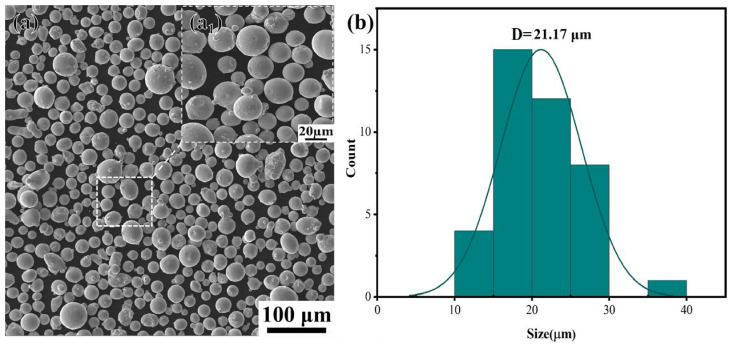
SEM image and particle size distribution diagram of the powder surface: (**a**) SEM image of the powder surface; (**a_1_**) Magnified image of powder morphplogy; (**b**) particle size distribution diagram of the powder.

The die-forged (DF) Inconel 718 plate and the raw Inconel 718 powder used for LPBF were both supplied by Hunan Hengji Powder Technology Co., Ltd. (Yueyang, China), with the powder having a particle size range of 15–53 μm. The SEM image and the particle size distribution of the powder are shown in Figure 1. The compositions of the LPBF alloy and the die-forged (DF) alloy are summarized in Table 1.

### 2.2. Sample Preparation and Heat Treatment

The die-forged (DF) alloy was fabricated using conventional casting + die forging (Shanghai Electric SHMP Casting & Forging Co., Ltd., Shanghai, China). The LPBF alloy was printed using a commercial FS191M LPBF machine which is manufactured by Farsoon Technologies Co., Ltd. (Changsha, China) with a laser power of 110 W, a scanning speed of 400 mm/s, a powder layer thickness of 30 μm, a 67° rotation scanning strategy, a spot diameter of 60 μm, and a scanning spacing of 0.08 mm. The scan strategy employs an 8 mm stripe pattern with a 67° rotation between successive layers, where each stripe is filled by parallel hatch lines. Argon gas was used to protect the alloy from oxidation during printing.

Both the die-forged (DF) and LPBF alloys were solution-treated at 1040 °C with a heating rate of 10 °C/min, held for 1 h, and subsequently water-quenched. The solution-treated samples are referred to as DF-ST and LPBF-ST, respectively. After the solution treatment, part of the DF and LPBF samples were age-treated at 700 °C for 48 h, also at a heating rate of 10 °C/min, followed by water quenching. The alloys subjected to both solution and aging treatment are designated as DF-STA and LPBF-STA, respectively.

### 2.3. Microstructure and Property Testing

The samples were processed into test pieces of Φ 6 × 9 mm along the XY direction using a wire electrical discharge machine (Shanghai Youtai Precise Machinery Co., Ltd., Shanghai, China). A Gleeble-3500 thermal simulation testing machine (Dynamic Systems Inc., Albany, NY, USA) was used to heat the test pieces to the deformation temperature T (450 °C, 550 °C, 650 °C, and 750 °C) at a rate of 10 °C/s, followed by holding for 5 min. The deformation rate (ε′) was 0.1 s^−1^, and the applied strain (ε) was 0.2 and 0.6. The compression direction is along the building (z) direction, and the subsequent microstructural characterization observation plane is also performed in the xoy plane. After compression deformation, the samples were immediately water-quenched to maintain their microstructure. The samples were sequentially ground with metallographic sandpaper ranging from 400 to 5000 mesh, then polished on the polishing machine. Finally, an HVS-1000 microhardness tester (Digital Display Vickers Microhardness Tester Model: HVS-1000, Shanghai Lidun Instrument & Meter Testing Technology Co., Ltd., Shanghai, China) was used to measure the Vickers microhardness. The applied load was 300 N, and the loading time was 15 s. For each sample, 10 microhardness points were tested, and the mean value is adopted. The etching solution used to reveal the microstructure consisted of 80 vol.% HCl, 13 vol.% HF, and 7 vol.% HNO_3_. The electrolytic polishing etching solution was 20 vol.% perchloric acid in alcohol solution, the electrolytic polishing working voltage was 25 V, the time was 25 s, and the electrolytic temperature was −25 °C. The electrolytically polished samples were placed in a JSM-7900F field emission electron microscope (JEOL Ltd., Tokyo, Japan) for EBSD characterization, and the data were analyzed using Channel 5 software. For TEM analysis, the samples were first ground to a thickness of approximately 90 μm, then punched into 3 mm diameter disks and thinned using a twin-jet electrolytic polisher (Suzhou Hanchu Optoelectronics Technology Co., Ltd., Suzhou, China). The electrolyte consisted of 7 vol.% perchloric acid and 93 vol.% ethanol, operated at an applied voltage of 25 V and a temperature of −25 °C.

## 3. Experimental Results and Discussions

### 3.1. Mechanical Behaviors Under Different Deformation Conditions

**Figure 2:**
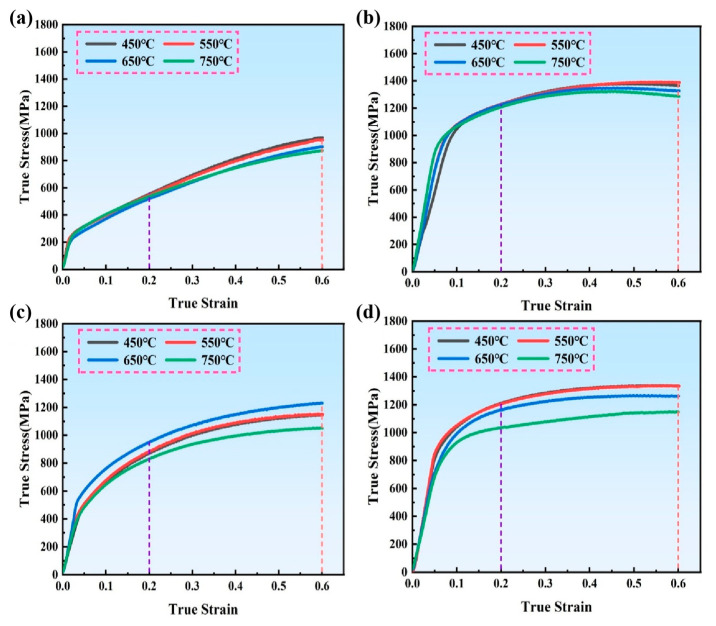
The true stress–strain curves of IN718 alloys under different deformation temperatures: (**a**) DF-ST alloy; (**b**) DF-STA alloy; (**c**) LPBF-ST alloy; (**d**) LPBF-STA alloy.

Figure 2 presents the true stress–true strain curves of the IN718 alloys deformed at various temperatures ranging from 450 °C to 750 °C, with the results summarized in Table 2. The DF and LPBF-built alloys under both ST and STA conditions exhibit a clear temperature-dependent mechanical response. For all samples, the flow stress decreases with increasing deformation temperature, reflecting the thermally activated softening behavior of IN718. Overall, the DF alloys (Figure 2a,b) display higher flow stress and greater work-hardening capability compared with the LPBF counterparts (Figure 2c,d). Moreover, the STA-treated alloys show significantly higher yield and peak stresses than the ST alloys. Notably, the difference between DF and LPBF alloys is more pronounced at lower deformation temperatures, suggesting that the deformation mechanisms of AM alloys are more sensitive to thermal activation and microstructural heterogeneity. Under the same deformation rate, it can be clearly seen that the flow stress shows a decreasing trend with the increase of temperature. This is because the increase in temperature enhances the thermal activation effect, increases the atomic kinetic energy, and reduces the critical shear stress of the slip system, resulting in a decrease in the deformation resistance of both alloys.

**Figure 3:**
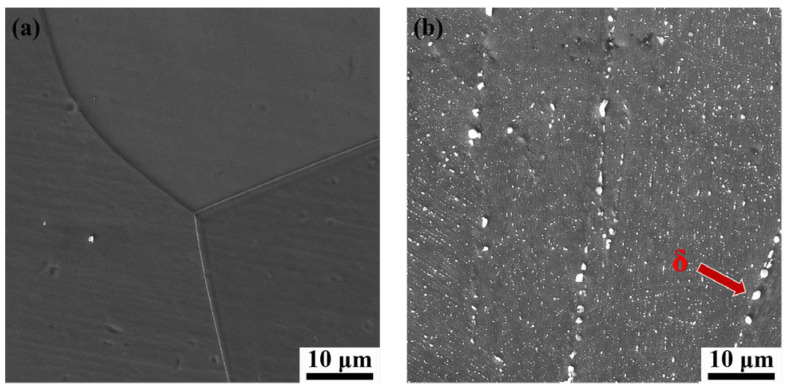
SEM microstructure before deformation: (**a**) DF-ST alloy; (**b**) LPBF-ST alloy.

Figure 3 presents the microstructures of the DF-ST and LPBF-ST alloys. Since the ST treatment (1040 °C) is much higher than the dissolution temperature of the second phases (γ″, γ′) in the DF alloy, there is no secondary phase in the DF-ST alloy. However, since the complete dissolution temperature of the δ phase in LPBF alloy is 1100 °C, short rod-like δ phases appear at the grain boundaries in the LPBF-ST alloy [18]. The δ phase appearing at the grain boundaries hinders the grain boundary sliding and improves the deformation resistance of the material. In addition, the tortuous grain boundaries also have a certain hindering effect on dislocations, so the flow stress of the LPBF alloy is greater than that of the DF alloy [34]. Further, after STA treatment, the flow stress of the IN718 alloy increases significantly. This is due to the fact that after the STA treatment, those strengthening phases precipitate from the alloy. The precipitation of the γ″ and γ′ phases can bring a large degree of misfit and coherent strain effect. Therefore, the fine precipitated phases hinder the dislocation slip during deformation, resulting in an increase in flow stress [35].

Notably, due to the presence of the δ phase, both the yield strength and compressive strength of the LPBF-ST alloy are higher than those of the DF-ST alloy, as shown in Table 2. However, the yield strength and compressive strength of the DF-STA alloy are higher than those of the LPBF-STA alloy. Owing to the existence of the γ″ and γ′ strengthening phases, the yield strength and compressive strength of both alloys after STA treatment are significantly improved at various deformation temperatures. Generally, the yield strength and compressive strength of the alloy decrease with the increase in deformation temperature. Nevertheless, the yield strength and compressive strength of the LPBF-ST alloy increase with the rise in temperature and reach a peak at 650 °C. This may be because the heat preservation treatment before deformation promotes the precipitation of the γ″ and γ′ strengthening phases in the matrix, thereby increasing its yield strength and compressive strength.

**Figure 4:**
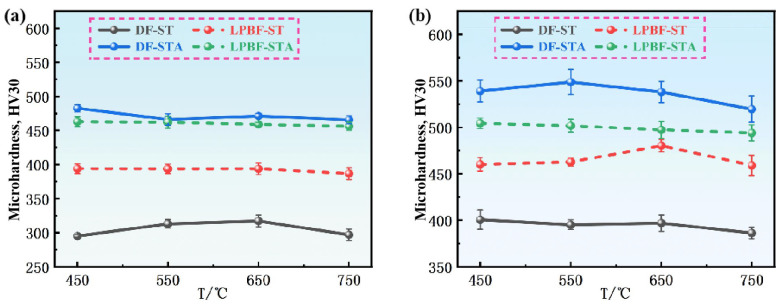
The Vickers microhardness of IN718 alloys deformed at different temperatures under two strains: (**a**) ε = 0.2; (**b**) ε = 0.6.

Figure 4 presents the Vickers microhardness of the IN718 alloys deformed at different temperatures under a true strain of 0.2 and 0.6, respectively. As the deformation temperature increases, the microhardness of the DF-STA, LPBF-ST, and LPBF-STA samples shows a gradual decrease when ε = 0.2, indicating thermal softening. In contrast, the DF-ST alloy exhibits a slight increase in microhardness followed by a decrease, reaching a peak value at 650 °C. At this strain level, the LPBF-ST alloy exhibits higher microhardness than the DF-ST alloy, whereas after STA treatment, the DF alloy shows superior hardness compared with its LPBF counterpart. This trend is consistent with the true stress–strain behavior shown in Figure 2. When the strain increases to 0.6, all alloys experience work hardening, leading to an overall increase in hardness. The LPBF-ST alloy reaches the highest hardness under this condition, while the general variation trends among the four alloys remain similar to those observed at ɛ = 0.2.

### 3.2. Metallographic Analysis and Grain Texture Evolution

**Figure 5:**
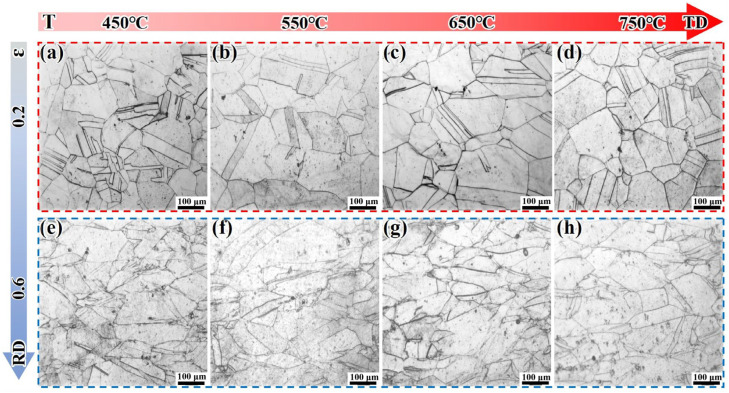
Microstructural evolution of the DF-ST alloy under different deformation conditions: (**a**–**d**) ε = 0.2; (**e**–**h**) ε = 0.6.

Figure 5 presents the microstructure of the DF-ST alloy under different deformation conditions. Under an ε of 0.2, the grains of the DF-ST alloy only undergo slight deformation, and the grain size increases with the increase in deformation temperature. When there is an ε of 0.6, the alloy grains are obviously elongated along the transverse direction (TD). With the increase in deformation temperature T, no DRX occurs. This may be due to the low dislocation density and low deformation storage energy inside the alloy, which are insufficient to trigger DRX.

**Figure 6:**
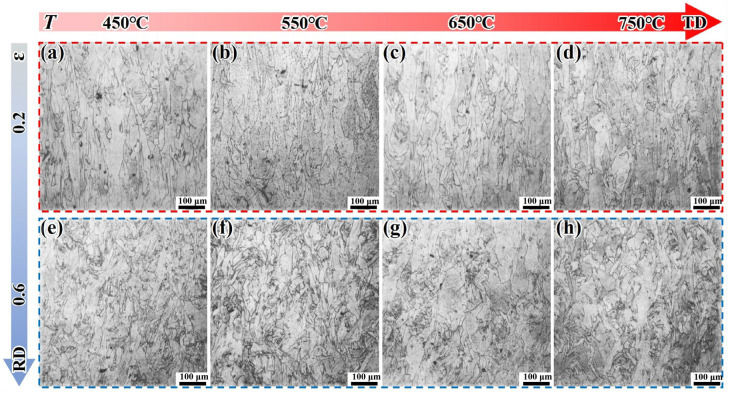
Microstructural evolution of the LPBF-ST alloy under different deformation conditions: (**a**–**d**) ε = 0.2; (**e**–**h**) ε = 0.6.

Figure 6 presents the microstructure of the LPBF-ST alloy under different deformation conditions. For the LPBF-ST alloy, under an ε of 0.2, the columnar grain structure shows no obvious variation with the increased deformation temperature. This may be because after ST treatment, δ phases precipitate at the grain boundaries, which inhibit grain growth. In addition, the bent columnar grain boundaries also have an inhibitory effect on dislocations. Under an ε of 0.6, the columnar grains exhibit obvious characteristics, such as epitaxial intersection and defect accumulation. Relatively dense small grains appear at the grain boundaries, and the tendency of the small grains to become more dense increases with the increased deformation temperature.

**Figure 7:**
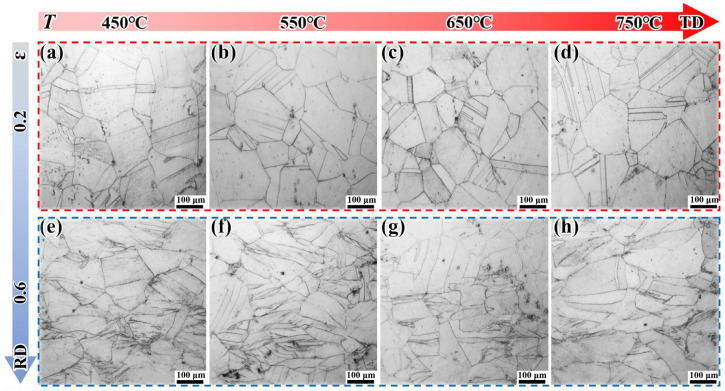
Microstructural evolutions of the DF-STA alloy under different deformation conditions: (**a**–**d**) ε = 0.2; (**e**–**h**) ε = 0.6.

Figure 7 presents the microstructure of the DF-STA alloy under different deformation conditions. After solution + aging treatment, the grain size of the DF alloy increases slightly. Under an ε of 0.2, it can be expected that a large number of secondary particles precipitate inside the grains. The grain structure does not change significantly with the increased deformation temperature. Under an ε of 0.6, the grains are obviously elongated along the TD direction, and characteristics such as defect accumulation appear. With the increase in temperature, the phenomenon of defect accumulation decreases, which may be due to the decrease in deformation resistance caused by the high-temperature softening.

**Figure 8:**
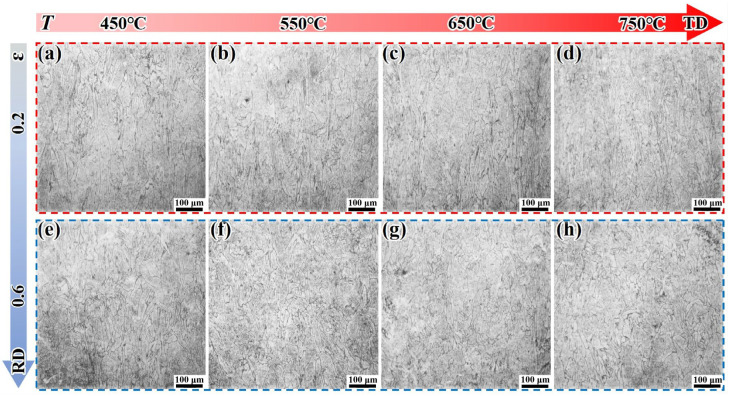
Microstructural evolution of the LPBF-STA alloy under different deformation conditions: (**a**–**d**) ε = 0.2; (**e**–**h**) ε = 0.6.

Figure 8 presents the microstructure of the LPBF-STA alloy under different deformation conditions. Under an ε of 0.2, the columnar grains are straight, and their uniformity is significantly better than that of the LPBF-ST alloy. The columnar grains tend to become finer and more densely distributed with the increase in temperature. Under an ε of 0.6, the grain shape does not change significantly with the increase in strain, but the degree of epitaxial cross-distribution of the columnar grains (Figure 6e–h) is significantly smaller than that of the LPBF-ST sample.

**Figure 9:**
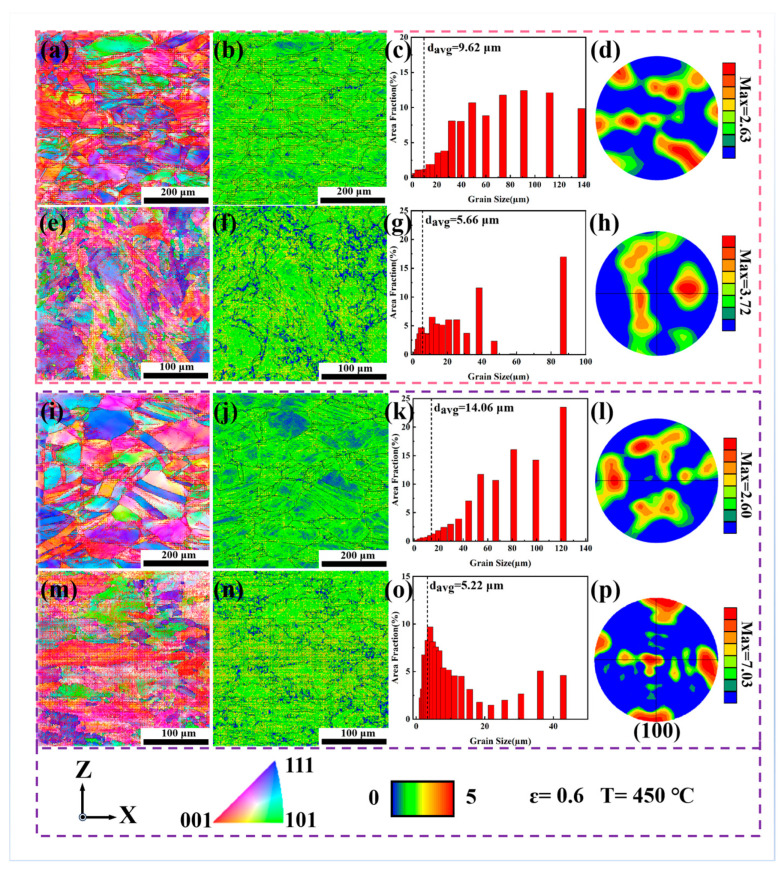
EBSD analysis of the IN718 alloys deformed at 450 °C (ε = 0.6): (**a**–**d**) DF-ST; (**e**–**h**) LPBF-ST; (**i**–**l**) DF-STA; (**m**–**p**) LPBF-STA.

**Figure 10:**
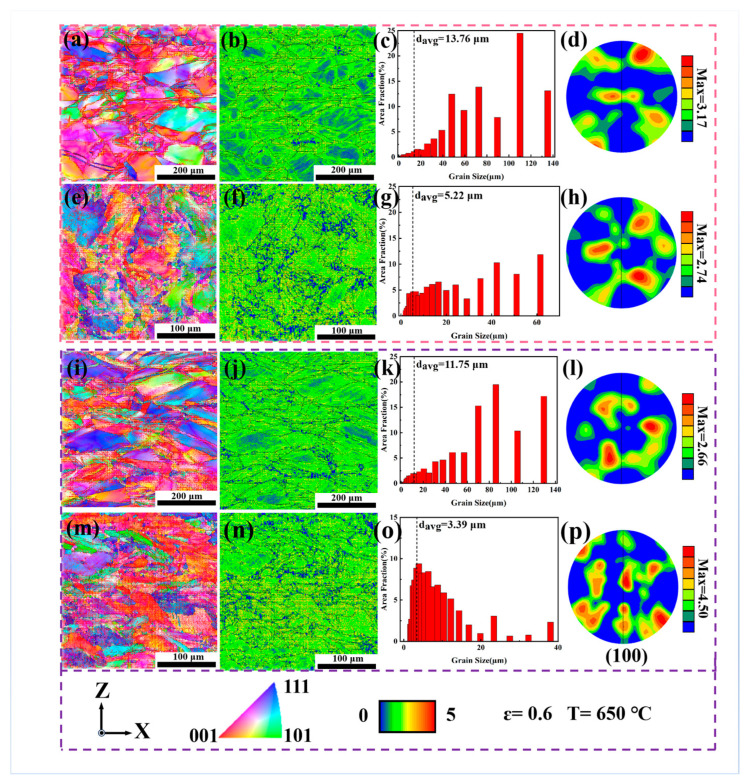
EBSD analysis of the IN718 alloys deformed at 650 °C (ε = 0.6): (**a**–**d**) DF-ST; (**e**–**h**) LPBF-ST; (**i**–**l**) DF-STA; (**m**–**p**) LPBF-STA.

To gain an in-depth understanding of the deformation mechanism of the alloys during intermediate-temperature deformation, EBSD analysis was conducted on the deformed samples. Figure 9 and Figure 10 present the EBSD results of the samples deformed at 450 °C and 650 °C, respectively (at a strain of ε = 0.6), including inverse pole figures (IPF), kernel average misorientation (KAM) maps, grain size distribution charts, and texture plots. At 450 °C, the KAM values at the grain boundaries and inside the grains of the DF-ST alloy are relatively consistent, indicating that the alloy deformation is uniform. In the LPBF-ST alloy, the dendritic grains and small grains are arranged along the building direction, and the KAM values inside the grains are relatively large. Only a few grains in the DF-STA alloy have small KAM values inside, and the KAM values at the grain boundaries and inside the grains are basically close, indicating relatively uniform alloy deformation. Notably, the LPBF-STA alloy has high KAM values inside the grains, which indicates that strengthening phases precipitate inside the grains, resulting in dislocation accumulation and concentrated stress distribution.

When the deformation temperature increases to 650 °C, the KAM values within the grains of the DF-ST alloy show a monotonically decreasing trend, which may be attributed to the reduced slip shear stress at higher temperatures. As the deformation temperature rises, the grain orientation gradually changes from the original <111> and <001> to predominantly <001>. The average grain size increases markedly, mainly consisting of large grains (d_avg > 60 μm), and the texture strength also increases to a certain degree of enhancement. In contrast, the KAM values within the grains of the LPBF-ST alloy increase slightly, which can be ascribed to the precipitation of the γ″ and γ′ strengthening phases inside the grains at 650 °C. This promotes dislocation accumulation and local stress concentration, leading to higher KAM values. Meanwhile, with an increasing deformation temperature, the grain orientation of the LPBF-ST alloy shifts mainly to <111> and <001>, the average grain size decreases (d___avg < 40 μm), and the texture strength is significantly decreased.

For the DF-STA alloy, the KAM values within the grains increase at 650 °C, likely because the γ″ strengthening phases precipitate inside the grains, which tend to transform into δ phases at this temperature. Consequently, the DF-STA alloy exhibits better deformation compatibility than the DF-ST alloy. In the LPBF-STA alloy, the KAM values at the grain boundaries decrease with rising temperature, while those within the grains remain relatively high, indicating reduced deformation uniformity. With increasing temperature, the grain orientation of the DF-STA alloy evolves mainly to <001>, the grains elongate along the TD direction, and the average grain size slightly decreases while remaining dominated by large grains (d_avg > 70 μm) with nearly unchanged texture strength, as presented in Table 3. In comparison, the LPBF-STA alloy develops a <001>-dominated texture, with a significant reduction in grain size and a dense distribution of fine grains (d_avg < 20 μm).

### 3.3. TEM Microstructural Analysis

**Figure 11:**
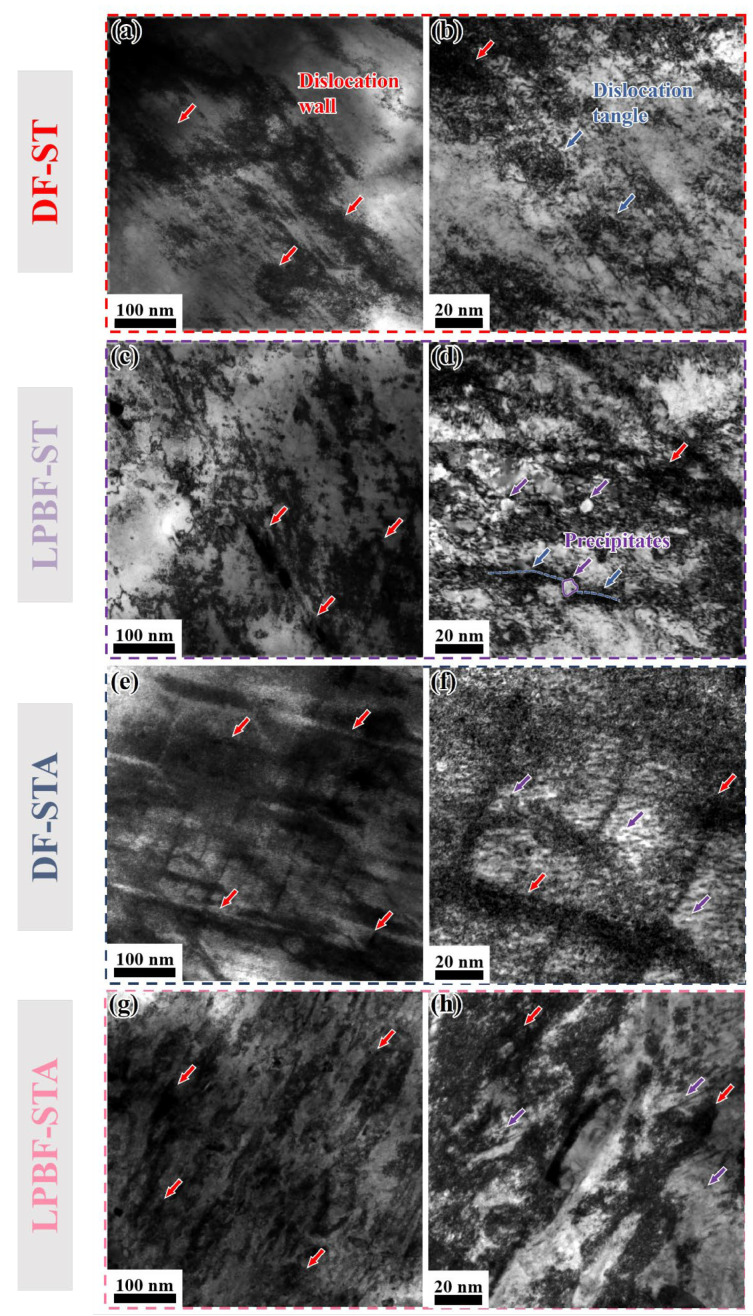
TEM analysis of the deformed heat-treated IN718 alloys (ε = 0.6): (**a**,**b**) DF-ST alloy; (**c**,**d**) LPBF-ST alloy; (**e**,**f**) DF-STA alloy; (**g**,**h**) LPBF-STA alloy. The red arrows indicate the distribution of dislocation walls, blue arrows denote the phenomenon of dislocation tangles, and purple arrows represent the precipitated second-phase particles.

Transmission electron microscopy (TEM) was used to analyze the microstructures of the deformed IN718 alloy, as shown in Figure 11. The DF-ST alloy only contains the γ matrix phase after solution treatment. The dislocation shows heterogeneous distribution, a large number of dislocation tangles appear, and its flow stress is approximately 969 MPa. In contrast, the LPBF-ST alloy still retains a small amount of the δ phase, and dislocation accumulation and tangling occur, thereby forming dislocation walls. In the LPBF-ST alloy, dislocations encounter precipitates of relatively large sizes, and these dislocations have a certain curvature. Its flow stress is about 1146 MPa, and the presence of the δ phase makes its flow stress higher than that of the DF-ST alloy. After STA treatment, the DF-STA alloy precipitates a large number of precipitated phases, resulting in more severe dislocation accumulation and tangling. The dislocations are obviously distributed along two different directions and are relatively uniform, with a flow stress of approximately 1390 MPa. A large number of precipitates can also be observed, and the dislocation density is higher. For the LPBF-STA alloy, the precipitated phases formed after aging treatment also led to the occurrence of dislocation accumulation and tangling. The dislocation walls increase significantly and are uniformly distributed, with a flow stress of about 1336 MPa, which is slightly lower than that of the DF-STA alloy (1390 MPa). This may be related to the more uniform distribution of dislocation accumulation and tangling in the DF-STA alloy. Its TEM images are basically consistent with the trend of the true stress–strain curve in Figure 2 and the hardness diagram in Figure 4.

## 4. Discussion

The comprehensive comparison between the LPBF-built and the DF IN718 alloys reveals that their mechanical behavior and deformation mechanisms are strongly governed by their distinct solidification and precipitation characteristics. The rapid solidification intrinsic to the LPBF process produces a fine columnar grain structure with pronounced elemental segregation and residual δ phase formation along the grain boundaries. In contrast, the DF alloy exhibits a relatively coarse and homogeneous equiaxed grain structure without a residual δ phase after solution treatment. These fundamental microstructural differences lead to markedly different strengthening behaviors, dislocation evolution, and deformation responses under intermediate-temperature conditions.

Under the ST condition, the LPBF alloy shows higher yield strength, compressive strength, and hardness than the DF alloy across the studied temperature range. This enhancement is mainly attributed to the finer columnar grains, higher dislocation density, and δ phase retention in the LPBF alloy, all of which impede dislocation motion and contribute to grain-boundary pinning. In contrast, the DF-ST alloy, with its coarser equiaxed grains and absence of the δ phase, exhibits a lower deformation resistance and more homogeneous strain distribution, as reflected by the relatively uniform KAM values.

After STA treatment, both alloys display substantial improvements in strength and hardness owing to the precipitation of γ″ and γ′ strengthening phases. However, the strengthening mechanisms differ between the two alloys. In the DF-STA alloy, the strengthening is dominated by the dense and uniform precipitation of the γ″/γ′ phases throughout the matrix, leading to a more homogeneous dislocation arrangement and higher flow stress. In the LPBF-STA alloy, the presence of residual δ phases and Nb segregation reduces the available Nb for γ″/γ′ formation, resulting in fewer strengthening precipitates and slightly lower strength. Moreover, the δ phase in the LPBF alloy effectively pins grain boundaries and refines the microstructure, but excessive δ precipitation can locally reduce ductility and lead to uneven deformation, as reflected by the higher KAM values within grains.

At intermediate temperatures (450–750 °C), the LPBF alloys exhibit more temperature-sensitive mechanical behavior than the DF alloys. Specifically, the LPBF-ST alloy reaches a peak in strength and hardness around 650 °C due to the temperature-induced precipitation of γ″/γ′ within the matrix, while the DF alloys demonstrate a monotonic decrease in strength with increasing temperature. The EBSD and TEM analyses further confirm that deformation in the DF alloys is characterized by uniform dislocation tangling and gradual texture evolution, whereas the LPBF alloys experience localized dislocation accumulation and stress concentration due to microsegregation and δ phase heterogeneity.

Overall, the LPBF-built IN718 alloy demonstrates higher strength in the solution-treated condition but slightly inferior mechanical properties after aging compared with the forged counterpart. The inherent δ phase retention, finer columnar grains, and Nb segregation in LPBF alloys critically alter their precipitation behavior and deformation mechanisms. This work provides mechanistic insight into how LPBF-induced microstructures—especially δ phase retention, Nb segregation, and columnar grain architecture—govern the intermediate-temperature deformation behavior of IN718. These findings directly inform industrial LPBF process optimization by identifying critical heat-treatment and microstructure-control strategies needed to match or surpass the performance of conventionally forged IN718. The demonstrated ability to tailor δ phase distribution and precipitation strengthening can guide the industrial design of LPBF-fabricated IN718 components for aerospace, energy, and high-temperature structural applications.

## 5. Conclusions

In this study, the intermediate-temperature deformation behaviors (450–750 °C) and underlying microstructural mechanisms of laser powder bed fusion (LPBF)-fabricated and conventionally die-forged (DF) IN718 alloy were systematically investigated and compared. The major conclusions can be summarized as follows:

(1) After ST treatment, the DF-ST alloy only contains the γ matrix, while the LPBF-ST alloy retains the δ phase at the grain boundaries. Under the ST condition, the LPBF alloy shows higher intermediate-temperature compressive strength than the DF alloy, primarily owing to grain refinement and δ phase pinning at grain boundaries. After STA treatment, both alloys are significantly strengthened by the γ″ and γ′ precipitates. However, in the LPBF alloy, Nb consumption by δ phase formation reduces γ″/γ′ precipitates, resulting in a slightly lower strength compared to the DF-STA alloy.
(2) The DF alloy shows a decreasing flow stress with increasing deformation temperature due to thermal activation and softening. However, the LPBF-ST alloy exhibits a peak in strength at around 650 °C, attributed to the temperature-induced precipitation of the γ″/γ′ phases within the matrix. EBSD and TEM analyses reveal that deformation in DF alloys is more homogeneous, while LPBF alloys exhibit localized dislocation accumulation and stress concentration arising from δ phase heterogeneity and microsegregation.
(3) The distinct precipitation and deformation behaviors between LPBF and DF alloys originate from their different solidification microstructures and Nb segregation characteristics. Controlling δ phase precipitation and homogenization heat treatment is crucial for balancing strength and ductility in additively manufactured IN718 superalloys.

## Figures and Tables

**Table 1:** Elemental composition table of die-forged alloy (DF) and additive manufacturing powder feedstock (LPBF) [33].

Element	Ni	Cr	Fe	Nb	Ti	Mo	Co	Al	Mn	Others
DF alloy	53.89	17.75	17.27	5.48	1.01	2.96	0.27	0.50	0.06	Bal.
LPBF alloy	53.24	19.41	15.76	5.48	0.95	3.26	0.92	0.42	0.28	Bal.

**Table 2:** Yield strength and compressive strength of IN718 alloy under different deformation conditions.

	Deformation Temperature (°C)	Yield Strength (MPa)	Compressive Strength (MPa)
DF-ST	450	240	969
LPBF-ST		405	1146
DF-STA		904	1390
LPBF-STA		853	1336
DF-ST	550	236	952
LPBF-ST		435	1152
DF-STA		899	1390
LPBF-STA		850	1336
DF-ST	650	208	902
LPBF-ST		540	1231
DF-STA		895	1347
LPBF-STA		721	1265
DF-ST	750	182	873
LPBF-ST		383	1050
DF-STA		890	1321
LPBF-STA		695	1150

**Table 3:** Grain size, texture intensity, and grain orientation of IN718 alloy under different deformation temperatures.

Alloy	Deformation Temperature (°C)	Grain Size (μm)	Texture Intensity	Grain Orientation
DF-ST	450	9.62	2.63	<001> <111>
DF-STA		14.06	2.60	<001> <111>
LPBF-ST		5.66	3.72	<001> <111>
LPBF-STA		5.22	7.03	<001> <101>
DF-ST	650	13.76	3.17	<001>
DF-STA		11.75	2.66	<001> <111>
LPBF-ST		5.22	2.74	<001> <111>
LPBF-STA		3.39	4.50	<001> <111>

## Data Availability

The original contributions presented in this study are included in the article. Further inquiries can be directed to the corresponding authors.
